# The Multiscale Surface Vision Transformer

**Published:** 2023-03-21

**Authors:** Simon Dahan, Abdulah Fawaz, Mohamed A. Suliman, Mariana da Silva, Logan Z. J. Williams, Daniel Rueckert, Emma C. Robinson

**Affiliations:** 1 Department of Biomedical Engineering, King’s College London; 2 Centre for the Developing Brain, King’s College London; 3 The Alan Turing Institute, London, United Kingdom; 4 Department of Computing, Imperial College London

**Keywords:** Deep Learning, Vision Transformers, Neuroimaging

## Abstract

Surface meshes are a favoured domain for representing structural and functional information on the human cortex, but their complex topology and geometry pose significant challenges for deep learning analysis. While Transformers have excelled as domain-agnostic architectures for sequence-to-sequence learning, notably for structures where the translation of the convolution operation is non-trivial, the quadratic cost of the self-attention operation remains an obstacle for many dense prediction tasks. Inspired by some of the latest advances in hierarchical modelling with vision transformers, we introduce the Multiscale Surface Vision Transformer (MS-SiT) as a backbone architecture for surface deep learning. The self-attention mechanism is applied within local-mesh-windows to allow for high-resolution sampling of the underlying data, while a shifted-window strategy improves the sharing of information between windows. Neighbouring patches are successively merged, allowing the MS-SiT to learn hierarchical representations suitable for any prediction task. Results demonstrate that the MS-SiT outperforms existing surface deep learning methods for neonatal phenotyping prediction tasks using the Developing Human Connectome Project (dHCP) dataset. Furthermore, building the MS-SiT backbone into a U-shaped architecture for surface segmentation demonstrates competitive results on cortical parcellation using the UK Biobank (UKB) and manually-annotated MindBoggle datasets. Code and trained models are publicly available at https://github.com/metrics-lab/surface-vision-transformers.

## 1 Introduction

In recent years, there has been an increasing interest in using attention-based learning methodologies in the medical imaging community, with the Vision Transformer (ViT) [8] emerging as a particularly promising alternative to convolutional methods. The ViT circumvents the need for convolutions by translating image analysis to a sequence-to-sequence learning problem, using self-attention mechanisms to improve the modelling of long-range dependencies. This has led to significant improvements in many medical imaging tasks, where global context is crucial, such as tumour or multi-organ segmentation [13, 15, 28]. At the same time, there has been a growing enthusiasm for adapting attention-based mechanisms to irregular geometries where the translation of the convolution operation is not trivial [11], but the representation of the data as sequences can be straightforward. For instance, transformer-based methodologies have been used in the context of molecular representations for modelling protein structure and interaction [2, 3, 16], or in the context of learning the spatio-temporal dynamics of functional connectomes [17]. Moreover, transformer models have emerged as a promising tool for modelling various cognitive processes. In particular, they have successfully captured the representation of spatial encoding in the hippocampus [30], as well as exhibiting brain-like representations of language and speech processing in transformer models trained on audiobooks [4, 23]. This highlights the potential of transformer models to bridge the gap between neural and computational models of cognition.

Despite promising results on high-level prediction tasks, one of the main limitations of the ViT remains the computational cost of the global self-attention operation, which scales quadratically with sequence length. This issue is particularly pronounced for high-resolution medical data. Various strategies have been developed to overcome this limitation, including restricting the computation of self-attention to local windows [10, 20] or implementing linear approximations [29, 31]. Among these, the hierarchical architecture of the Swin Transformer [20] has emerged as a particularly promising candidate. This implements windowed-self-attention, alongside a shifted window strategy that allows cross-window connections. Neighbouring patch tokens are progressively merged across the network, producing a hierarchical representation of image features. This hierarchical strategy has shown to improve performance over the global-attention approach of the standard ViT, and has already found applications within the medical imaging domain [13].

In this paper, we therefore introduce the Multiscale Surface Vision Transformer (MS-SiT) as a novel backbone architecture for surface deep learning. The MS-SiT extends the global attention-based approach of the Surface Vision Transformer (SiT) [6], to a hierarchical version that can serve as a backbone for any high-level or dense prediction task on sphericalised meshes. We evaluate our approach on neonatal phenotype prediction tasks derived from the Developing Human Connectome Project (dHCP), as well as on cortical parcellation for both UK Biobank (UKB) and manually-annotated MindBoggle datasets. Key contributions are summarised as follows:

- We introduce the Multiscale Surface Vision Transformer (MS-SiT), a fully-attention-based framework for surface deep learning, implementing hierarchical feature aggregation on sphericalised meshes.
- Our proposed MS-SiT architecture surpasses existing surface deep learning methods for predictions of cortical phenotypes and achieves competitive performance on cortical parcellation tasks, highlighting its potential as a powerful tool for clinical applications.
- Performance is further enhanced through the translation of the Shifted Window Multi-Head Self-Attention mechanism [20] to genus-zero surface domains.

## 2 Methods

### 2.1 Architecture overview

#### Backbone

**Fig. 1:**
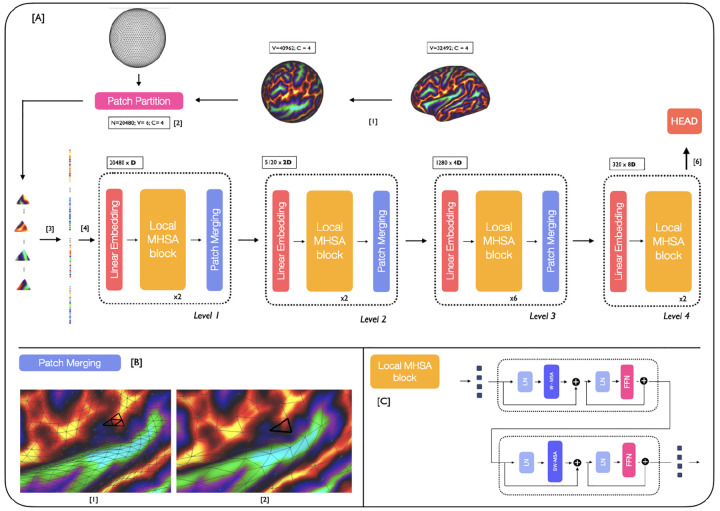
[A] MS-SiT pipeline. The input cortical surface is resampled from native resolution to an ico6 input mesh (1) and partitioned, using an ico5 grid, into a sequence of 20480 non-overlapping triangular patches (2). The sequence is then flattened (3) and passed to the MS-SiT encoder layers (4). At the end of the encoder, (5) the head can be adapted for classification or regression tasks. [B] An example of the patch merging process from *I*_4_ to *I*_3_ grid. [C] Two local-MHSA blocks applying successively with W-MHSA and SW-MHSA.

The proposed MS-SiT adapts the Swin Transformer architecture [20] to the case of cortical surface analysis, as illustrated in Figure 1. Here, input data $X\in {\mathbb{R}}^{\left|V_{6}\right|\times C}$ (*C* channels) is represented on a 6th-order icospheric (ico6) tessellation: *I*_6_ = (*V*_6_, *F*_6_), with |*V*_6_| = 40962 vertices and |*F*_6_| = 40962 faces. This data is first partitioned into a sequence of |*F*_5_| = 20480 non-overlapping triangular patches: $T_{5}=\left\{t_{5}^{1},t_{5}^{2},\;..t_{5}^{\left|F_{5}\right|}\right\}$ (with $t_{5}^{i}\subset V_{6}$, $\left|t_{5}^{i}\right|=6$), by patching the data with ico5: *I*_5_ = (*V*_5_, *F*_5_), |*V*_5_| = 10242, |*F*_5_| = 20480 (Figure 1 A.2). Imaging features for each patch are then concatenated across channels, and flattened to produce an initial sequence: $X^{0}=\left[X_{1}^{0},\;\ldots ,X_{\left|F_{5}\right|}^{0}\right]\in {\mathbb{R}}^{\left|F_{5}\right|\times \left(\left|t_{5}\right|C\right)}$ (Figure 1A.3). Positional embeddings, LayerNorm (LN) and a dropout layer are then applied, before passing it to the MS-SiT encoder, organised into *l* = {1, 2, 3, 4} levels.

At each level of the encoder, a linear layer projects the input sequence *X*^*l*^ to a 2^(*l*−1)^ × *D*-dimensional embedding space: $X_{\text{emb}}^{l}\in {\mathbb{R}}^{\left|F_{6-l}\right|\times 2^{\left(l-1\right)}D}$. Local multi-head self-attention blocks (local-MHSA), described in detail below, are then applied, outputting a transformed sequence of the same resolution $\left(X_{\text{MHSA}}^{l}\in {\mathbb{R}}^{\left|F_{6-l}\right|\times 2^{\left(l-1\right)}}D\right)$. This is subsequently downsampled through a patch merging layer, which follows the regular downsampling of the icosphere, to merge clusters of 4 neighbouring triangles together (Figure 1B.), generating output: $X_{\text{out}}^{l}\in {\mathbb{R}}^{\left|F_{6-l-1}\right|\times 2^{\left(l+1\right)}D}$.

This process is repeated across several layers, with the spatial resolution of patches progressively downsampled from *I*_5_ → *I*_4_ → *I*_3_ → *I*_2_, but the channel dimension doubling each time. In doing so, the MS-SiT architecture produces a hierarchical representation of patch features, with respectively |*F*_5_| = 20480, |*F*_4_| = 5120, |*F*_3_| = 1280, and |*F*_2_| = 320 patches. In the last MS-SiT level, the patch merging layer is omitted and the sequence of patches is averaged into a single token, and input to a final linear layer, for classification or regression (Figure 1A.5). The segmentation pipeline employs a UNet-like architecture, with skip-connections between encoder and decoder layers, and patch partition instead of patch merging applied during decoding. An illustration of the segmentation architecture is provided in the Supplementary (Figure S.1).

#### Local Multi-Head Self-Attention blocks

Local Multi-Head Self-Attention blocks are defined similarly to ViT blocks [8]: as successive multi-head self-attention (MHSA) and feed-forward (FFN) layers, with LayerNorm (LN) and residual layers in between (Figure 1C). Here, a **W**indow-MHSA (**W-MHSA**) replaces the global MHSA of standard vision transformers, applying self-attention between patches within non-overlapping local mesh-windows. To provide the model with sufficient contextual information, this attention window is defined by an icosahedral tessellation two levels down from the resolution used to represent the feature sequence. This means that at level *l*, while the sequence is represented by *I*_6−*l*_, the attention windows correspond to the non-overlapping faces *F*_6−(*l*+2)_ defined by *I*_6−(*l*+2)_. For example, at level 1 the features are input at ico5, and local attention is calculated between the subset of 64 triangular patches that overlap with each face of ico3 (*F*_3_). Only in the last layer, is attention not restricted to local windows but applied globally to the *I*_2_ grid, allowing for global sharing of information across the entire sequence. More details of the parameterisation of window attention grids is provided in Supplementary Table S.1. This use of local self-attention significantly reduces the computational cost of attention at level *l*, from 𝒪(|*F*_6−*l*_|^2^) to 𝒪(*w*_*l*_|*F*_6−*l*_|) with *w*_*l*_ ≪ |*F*_6−*l*_|.

#### Self-Attention with Shifted Windows

Cross-window connections are introduced through **S**hifted **W**indow MHSA (**SW-MHSA**) modules, in order to improve the modelling power of the local self-attention operations. These alternate with the W-MHSA, and are implemented by shifting all the patches in the sequence *I*_6−*l*_, at level *l*, by *w*_*s*_ positions, where *w*_*s*_ is a fraction of the window size *w*_*l*_ (typically *w*_*l*_ = 64). In this way, a fraction of the patches of each attention window now falls within an adjacent window. This preserves the cost of applying self-attention in a windowed fashion, whilst increasing the models representational power by sharing information between non-overlapping attention windows. An illustration of two successive local-MHSA blocks implementing W-MHSA and SW-MHSA is provided in Figure 1C. Implementation can be summarised as follows:

(1) $${\hat{\text{X}}}^{l}=W-MSA\left({\text{X}}_{\text{emb}}^{l}\right)+{\text{X}}_{\text{emb}}^{l}\;{\text{Z}}^{l}=FFN\left({\hat{\text{X}}}^{l}\right)+{\hat{\text{X}}}^{l}\;{\hat{\text{Z}}}^{l}=SW-MSA\left({\text{Z}}^{l}\right)+{\text{Z}}^{l}\;{\text{X}}_{\text{MHSA}}^{l}=FFN\left({\hat{\text{Z}}}^{l}\right)+{\hat{\text{Z}}}^{l}$$

Here ${\text{X}}_{\text{emb}}^{l}$ and ${\text{X}}_{\text{MHSA}}^{l}$ correspond to input and output sequences of the local-MHSA block at level *l*. Residual connections are referred to by the + symbol.

#### Training details

Augmentation strategies were introduced to improve regularisation and increase transformation invariance. This included implementing random rotational transforms, where the degree of rotation about each axis was randomly sampled in the range ∈ [−30°, +30°] (for the regression tasks) and ∈ [−15°, +15°] (for the segmentation tasks). In addition, elastic deformations were simulated by randomly displacing the vertices of a coarse ico2 grid to a maximum of 1/8th of the distance between neighbouring points (to enforce diffeomorphisms [11]). These deformations were interpolated to the high-resolution grid of the image domain, online, during training. The effect of tuning the parameters of the SW-MHSA modules is presented in the Supplementary Table S.2 and reveals that the best results are obtained while shifting half of the patches.

## 3 Experiments & Results

All experiments were run on a single RTX 3090 24GB GPU. The AdamW optimiser [21] with Cosine Decay scheduler was used as the default optimisation scheme. A combination of Dice Loss and CrossEntropyLoss was used for the segmentation tasks and MSE loss was used for the regression tasks. Surface data augmentation was randomly applied with a probability of 80%. If selected, one random transformation is applied: rotation (50%) or non-linear warping (50%). For all regression tasks, a custom balancing sampling strategy was applied to address the imbalance of the data distribution.

### 3.1 Phenotyping predictions on dHCP data

#### Data

Data from the dHCP comes from the publicly available third release^5^ [9] and consists of cortical surface meshes and metrics (sulcal depth, curvature, cortical thickness and T1w/T2w myelination) derived from T1- and T2-weighted magnetic resonance images (MRI), using the dHCP structural pipeline, described by [22] and references therein [5, 14, 19, 22, 27]. In total 580 scans were used from 419 term neonates (born after 37 weeks gestation) and 111 preterm neonates (born prior to 37 weeks gestation). 95 preterm neonates were scanned twice, once shortly after birth, and once at term-equivalent age.

#### Tasks and experimental set up:

Phenotype regression was benchmarked on two tasks: prediction of postmenstrual age (PMA) at scan, and gestational age (GA) at birth. Here, PMA was seen as a model of ‘healthy’ neurodevelopment, since training data was drawn from the scans of term-born neonates and preterm neonates’ first scans: covering brain ages from 26.71 to 44.71 weeks PMA. By contrast, the objective of the GA model was to predict the degree of prematurity (birth age) from the participants’ term-age scans, thus the model was trained on scans from term neonates and preterm neonates’ second scans. Experiments were run on both ***template***-aligned data and unregistered (***native***) to evaluate the robustness of surface deep learning methods on non-aligned data and compared against surface convolutional approaches (Spherical UNet (SUNet) [32] and MoNet [24]). Training test and validation sets were allocated in the ratio of 423:53:54 examples (for PMA) and 411:51:52 (for GA) with a balanced distribution of examples, within each partition, from each age bin.

#### Results

Results from the phenotyping prediction experiments are presented in Table 1, where the MS-SiT models were compared against several surface convolutional approaches and three versions of the Surface Vision Transformer (SiT) using different grid sampling resolutions. The MS-SiT model consistently outperformed all other models across all prediction tasks (PMA and GA) and data configurations (template and native). Specifically, on the PMA task, the MS-SiT model outperformed other models by over 54% compared to Spherical UNet [32], 13% to MoNet [24], and 12% to the SiT (ico3) (average over both data-configurations), achieving a prediction error of 0.49 MAE on template data, which is within the margin of error of age estimation in routine ultrasound (typically, 5 to 7 days on average). On the GA task, the MS-SiT model achieved an even larger improvement with 49%, 43%, and 21% reduction in MAE relative to Spherical UNet, MoNet, and SiT (ico3), respectively. Importantly, the model demonstrated much greater transformation invariance, with only a 5% drop in performance between the template and native configurations, compared to 53% for Spherical UNet, and 10% for MoNet. Results also revealed a significant benefit to using the SW-MHSA with a 16% improvement over the vanilla version on GA predictions.

### 3.2 Cortical parcellation on UKB & MindBoggle

#### Data & Tasks

Cortical segmentation was performed using 88 manually labelled adult brains from the MindBoggle-101 dataset [18], annotated into 32 regions following a modified version of the Desikan–Killiany–Tourville (DKT) [18] pipeline, which delineates regions according to features of cortical shape. Surface files were processed with the CIFTIFY pipeline [7], which implements HCP-style post-processing including file conversion to GIFTI and CIFTI formats, and MSM Sulc alignment [25, 26]^6^. Separately, FreeSurfer annotation parcellations (based on a standard version of the DK atlas with 35 regions) were available for 4000 UK Biobank subjects, processed according to [1]. These were used for pretraining. In both cases, datasets were split into 80/10/10 sets. Sulcal depth and curvature maps were used as input features.

#### Results

**Fig.2:**
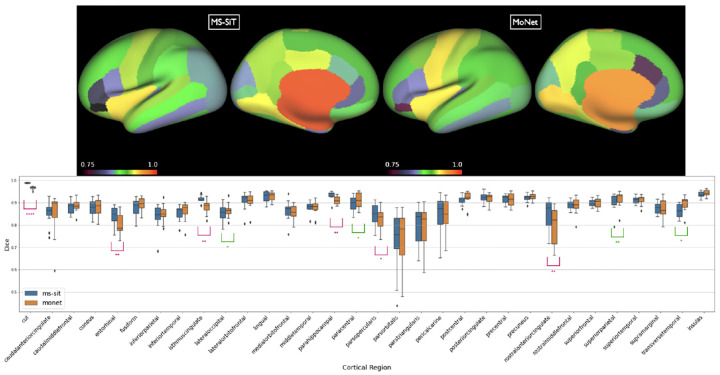
Top: Inflated surface showing mean dice scores shown for each of the DKT regions, for both MoNet and the pre-trained MS-SIT. Bottom: Boxplots comparing regional parcellation results. Asterisks denote statistical significance for one-sided paired t-test (pink: MS-SiT > MoNet; green MoNet > MS-SIT; ****: *p* < 0.0001, **: *p* < 0.01, *: *p* < 0.05).

Results are presented in Table 2. The Multiscale Surface Vision Transformer (MS-SiT) was compared against two other geometric deep learning approaches for cortical segmentation: Adv-GCN, a graph-based method optimized for alignment invariance [12], and MoNet, which learns filters by fitting mixtures of Gaussians on the surface [24]. MoNet achieved the best dice results overall. However, a per region box plot comparison (Fig 2) of its performance relative to the MS-SIT (pre-trained with Biobank) shows this is largely driven by improvements to two large regions. Overall, MoNet and the MS-SIT differ significantly for 10 out of 32 regions, with MS-SIT outperforming MoNet for 6 of these.

## 4 Discussion

The novel MS-SiT network presents an efficient and reliable framework, based purely on self-attention, for any biomedical surface learning task where data can be represented on sphericalised meshes. Unlike existing convolution-based methodologies translated to study general manifolds, MS-SiT does not compromise on filter expressivity, computational complexity, or rotational equivariance [11]. Instead, with the use of local and shifted attention, the model is able to effectively reduce the computational cost of applying attention on larger sampling grids, relative to [6], improving phenotyping performance, and performing competitively on cortical segmentation. Compared to convolution-based approach, the use of attention allows for the retrieval of attention maps, providing interpretable insights into the most attended cortical regions (Supplementary S.2), and the methodology’s invariance to transformations enables it to perform well on both aligned and non-aligned data, removing the need for pre-alignment.

## Supplementary Material

1

## Figures and Tables

**Table 1:** Test results for the dHCP phenotype prediction tasks: PMA and GA. **M**ean **A**bsolute **E**rror (MAE) and std are averaged across three training runs for all experiments.

Model	Aug.	Shifted Attention	PMA Template	PMA Native	GA Template	GA Native
SUNet [32]	✔	n/a	0.75±0.18	1.63±0.51	1.14±0.17	2.41±0.68
MoNet [24]	✔	n/a	0.61±0.04	0.63±0.05	1.50±0.08	1.68±0.06
SiT-T (ico2)	✔	n/a	0.58±0.02	0.66±0.01	1.04±0.04	1.28±0.06
SiT-T (ico3)	✔	n/a	0.54±0.05	0.68±0.01	1.03±0.06	1.27±0.05
SiT-T (ico4)	✔	n/a	0.57±0.03	0.83±0.04	1.41±0.09	1.49±0.10
MS-SiT	✔	✘	**0.49**±**0.01**	**0.59**±**0.01**	1.00±0.04	1.17±0.04
MS-SiT	✔	✔	**0.49**±**0.01**	**0.59**±**0.01**	**0.88**±**0.02**	**0.93**±**0.05**

**Table 2:** Overall mean and standard deviation of Dice scores (across all regions).

Methods	Aug.	Shifted Attention	Dice
Adv-GCN^7^[12]	n/a	n/a	0.857±0.035
MoNet [24]	✔	n/a	**0.910**±**0.01**
MS-SiT	✔	✔	0.897±0.01
MS-SiT (UKB)	✔	✔	0.901±0.01

## Footnotes

5 http://www.developingconnectome.org.

6 13 datasets failed due to missing files

7 run on a different train/test split
