# Supplementary material for: The Multiscale Surface Vision Transformer
Source: ArXiv. 2023 Mar 21:arXiv:2303.11909v1. Preprint. [Version 1] (PMC10055498)
Supplement: Supplementary file 1 [file NIHPP2303.11909V1-supplement-1.pdf]

A Supplementary material

MS-SiT levels	Sequence length $\|F_{6-l}\|$	Nbr attention windows	Window size w_l	MS-SiT
$l=1$	20480	320	64	linear 96-d - LN<br>$\begin{bmatrix} w_l \text{ 64} \\ \text{dim 96, head 3} \end{bmatrix} \times 2$<br>merging - LN
$l=2$	5120	80	64	linear 192-d - LN<br>$\begin{bmatrix} w_l \text{ 64} \\ \text{dim 192, head 6} \end{bmatrix} \times 2$<br>merging - LN
$l=3$	1280	20	64	linear 384-d - LN<br>$\begin{bmatrix} w_l \text{ 64} \\ \text{dim 384, head 12} \end{bmatrix} \times 6$<br>merging - LN
$l=4$	320	1	320	linear 768-d - LN<br>$\begin{bmatrix} w_l \text{ 64} \\ \text{dim 768, head 24} \end{bmatrix} \times 2$<br>merging - LN

Table S.1: MS-SiT detailed architecture. The MS-SiT model adapts the Swin-T architecture [20], into a 4-level network with $\{2, 2, 6, 2\}$ local-MHSA blocks and $\{3, 6, 12, 24\}$ attention heads per level. As in [20], the initial embedding dimension is $D = 96$. Thus, the MS-SiT encoder has 29.5 M trainable parameters.

**Shifted attention**	**Shift factor w_s**	**MAE**	**MAE (augmentation)**
✗	✗	1.68 ± 0.13	1.24 ± 0.04
✓	1/2	1.55 ± 0.03	**1.16 ± 0.02**
✓	1/4	**1.54 ± 0.08**	1.20 ± 0.03
✓	1/16	1.56 ± 0.09	1.26 ± 0.04

Table S.2: Hyper-parameter tuning of the shift factor w_s in the **SW-MHSA** module. The use of $w_s \in \{\frac{1}{2}, \frac{1}{4}, \frac{1}{16}\}$ is compared to no shift for MS-SiT models trained on GA-template and for 25k iterations ($\sim$ 50% typical training runtime). MAE and std on validation data, averaged over 3 runs are reported. Shifting the sequence of half the length of the attention windows, i.e. $w_s = \frac{1}{2}$, provides the best results overall and is used in all the following.

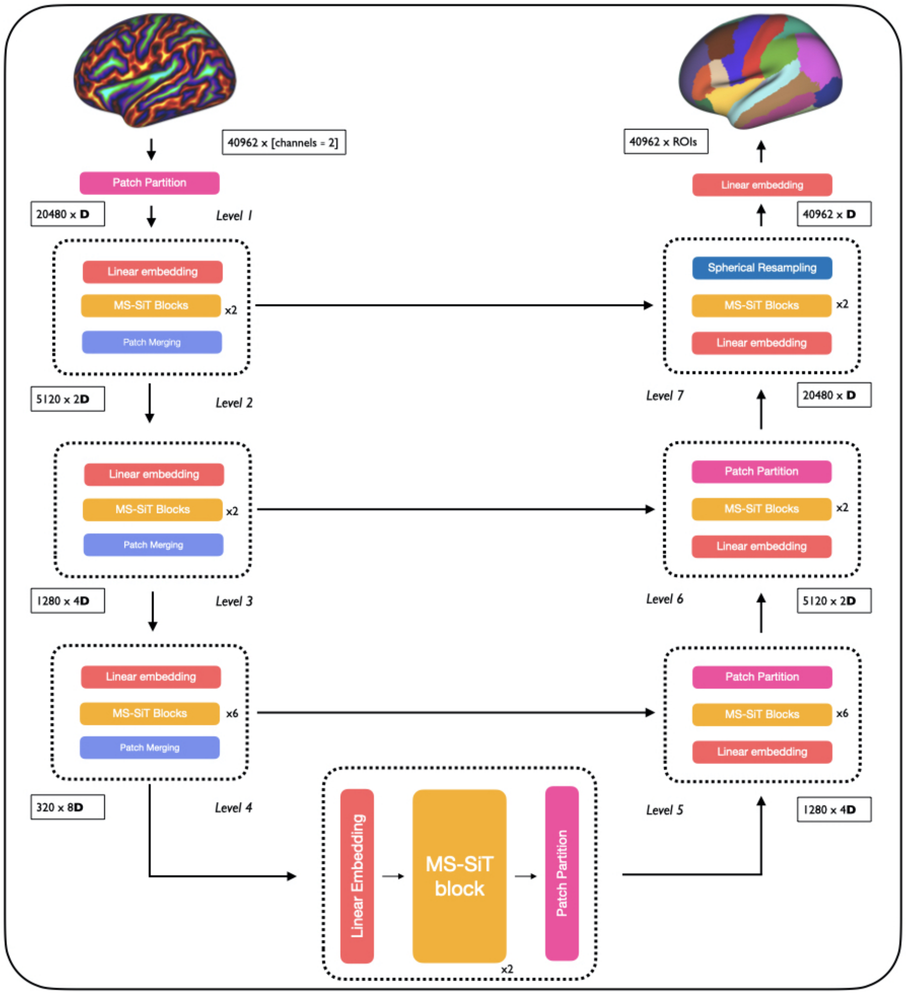

Fig. S.1: MS-SiT segmentation pipeline. Input data is resampled and partitioned as in Figure 1. The $l = \{1, 2, 3, 4\}$ levels of the segmentation pipeline are similar to the MS-SiT encoder levels (Figure 1). The *patch partition* layers reverse the patch merging procedure of the MS-SiT encoder, upsampling the spatial resolution of patches from $I_2 \rightarrow I_3 \rightarrow I_4 \rightarrow I_5$. Skip connections between levels are used. Finally, a *spherical resampling* layer resamples the final embeddings to an ico6 tessellation (40962 vertices), before the final segmentation prediction.

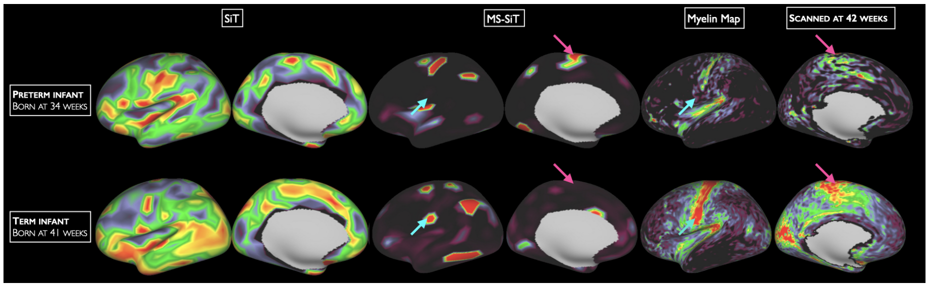

Fig. S.2: Comparison of normalised attention maps from the last layers of a SiT model [11] (applying global attention for all layers) and an MS-SiT model, both trained for GA-template prediction. MS-SiT maps display highly specific attention patterns, compared to the SiT counterparts, focusing on characteristic landmarks of cortical development such as the sensorimotor cortex with low myelination in preterm (pink arrows) and high myelination in term (blue arrows).
